# 167. Phase 3, Randomized, Controlled Trial Evaluating Safety, Efficacy, and Pharmacokinetics (PK) of Clesrovimab in Infants and Children at Increased Risk for Severe Respiratory Syncytial Virus (RSV) Disease

**DOI:** 10.1093/ofid/ofae631.004

**Published:** 2025-01-29

**Authors:** Heather J Zar, Louis J Bont, Paolo Manzoni, Flor M Munoz, Octavio Ramilo, Po-Yen Chen, Jose M Novoa Pizarro, Gustavo A Ordonez, Maria Tsolia, Bruce Tapiero, Mirta Acuña, Javier M Castellanos, Michael Meyer, Ichiro Morioka, Ziqiang Chen, Radha A Railkar, Xiaowei Zang, Andrea L Krick, Andrew W Lee, Luis A Castagnini, Anushua Sinha

**Affiliations:** Red Cross Children's Hospital and SA-MRC Unit on Child & Adolescent Health, University of Cape Town, South Africa, Cape Town, Western Cape, South Africa; University Medical Centre Utrecht, Zeist, Utrecht, Netherlands; University of Torino, Torino, Piemonte, Italy; Baylor College of Medicine, Houston, TX; St. Jude Children's Research Hospital, Memphis, TN; Taichung Veterans General Hospital, Taichung, Taitung, Taiwan; Hospital Padre Alberto Hurtado, Santiago, Region Metropolitana, Chile; Univeridad Del Valle, Cali, Valle del Cauca, Colombia; National and Kapodistrian University of Athens, Athens, Attiki, Greece; CHU Sainte-Justine, Montreal, Quebec, Canada; Roberto del rio children hospital, Santiago, Region Metropolitana, Chile; Morales Vargas Centro de Investigacion, Leon, Guanajuato, Mexico; Middlemore Hospital, Auckland, Auckland, New Zealand; Nihon University Itabashi Hospital, Tokyo, Tokyo, Japan; Merck & Co., Inc., Rahway, New Jersey; Merck & Co., Inc., Rahway, New Jersey; Merck & Co., Inc., Rahway, New Jersey; Merck & Co., Inc., Rahway, New Jersey; Merck & Co., Inc., present affiliation Uniquity Bio, Malvern, PA, USA, Rahway, New Jersey; Merck & Co., Inc., Rahway, New Jersey; Merck and Co Inc., Rahway, NJ

## Abstract

**Background:**

Clesrovimab is an investigational, long-acting monoclonal antibody for the prevention of RSV lower respiratory tract infection (LRI) in infants, including those at high risk of severe RSV disease due to serious comorbidity or premature birth.

**Figure:**
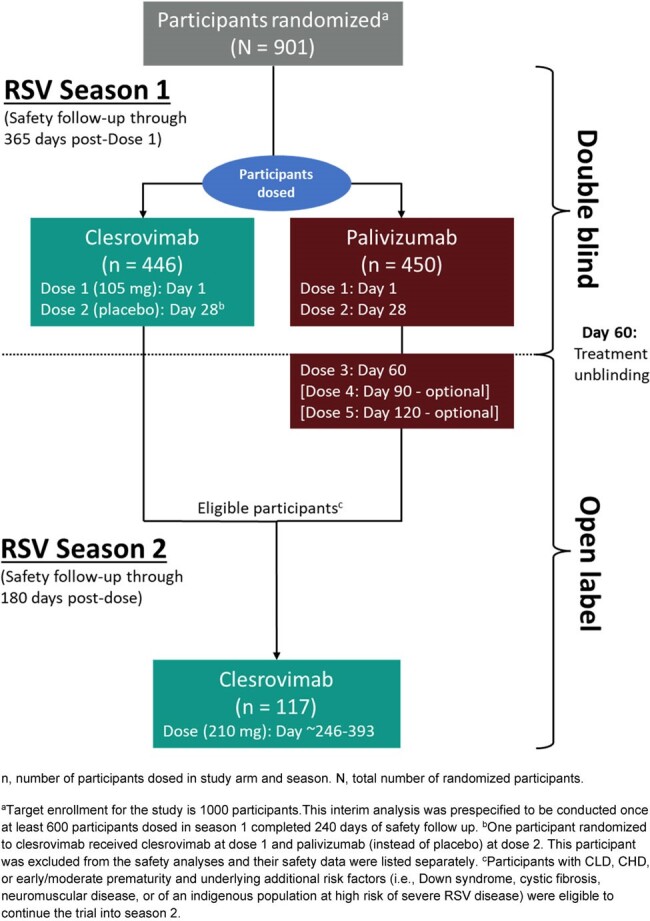

**Methods:**

This is a planned interim analysis (IA) of a randomized, controlled, phase 3 trial in infants entering their first RSV season recommended to receive palivizumab due to prematurity (≤35 weeks gestational age), chronic lung disease (CLD) of prematurity, or hemodynamically significant congenital heart disease (CHD). Participants (pts) were randomized 1:1 to receive clesrovimab (105 mg IM on day 1, placebo on day 28) or monthly palivizumab in season 1; eligible pts received clesrovimab (210 mg IM) in season 2 (Figure 1). The primary endpoint was safety and tolerability of clesrovimab vs. palivizumab in season 1. Secondary endpoints included the incidence of RSV-associated medically attended LRI (MALRI) requiring ≥1 indicator of LRI or severity and of RSV-associated hospitalization through day 150. Clesrovimab serum PK was analyzed through day 150.

**Figure:**
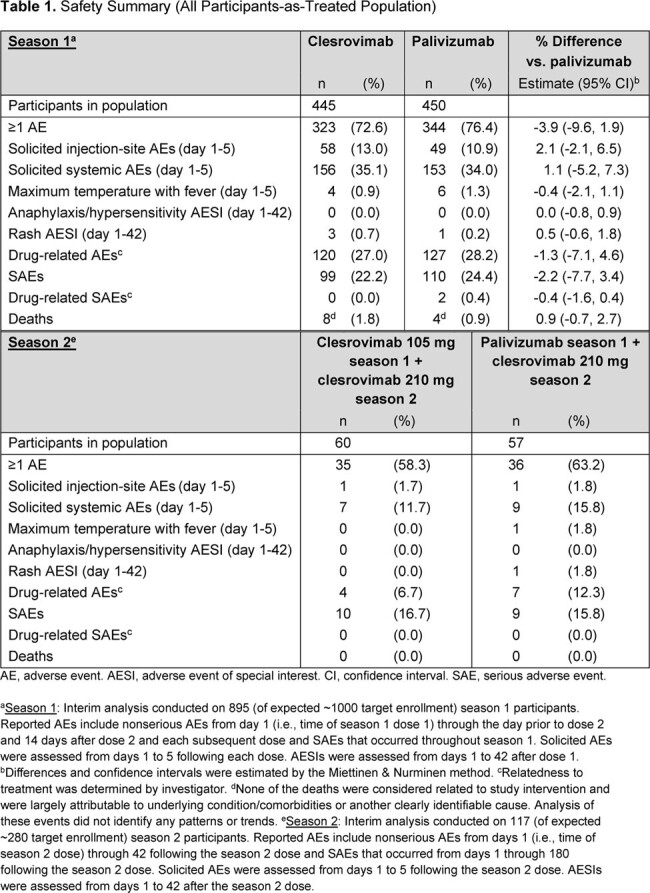

**Results:**

At this IA, 901 pts had been randomized into the trial. Baseline characteristics were well balanced; 28% had CLD, 11% had CHD, and 61% were born preterm without CLD/CHD. In season 1, the proportion of pts with AEs were comparable between arms; no pts in the clesrovimab arm had a drug-related serious AE (Table 1). In the season 2 IA, proportions of pts with AEs were comparable between those who had received clesrovimab or palivizumab in season 1. There were 8 deaths (1.8%) in the clesrovimab and 4 (0.9%) in the palivizumab arm, all attributable to underlying comorbidities or causes unrelated to treatment. No anaphylaxis/hypersensitivity reactions were reported. Incidence rates of RSV-associated MALRI and of RSV-associated hospitalization were comparable between clesrovimab (3.6% and 1.3%, respectively) and palivizumab (3.0% and 1.5%, respectively) through day 150 (Table 2). In season 1, the geometric mean half-life of clesrovimab was 44.1 days (Table 3).

**Figure:**
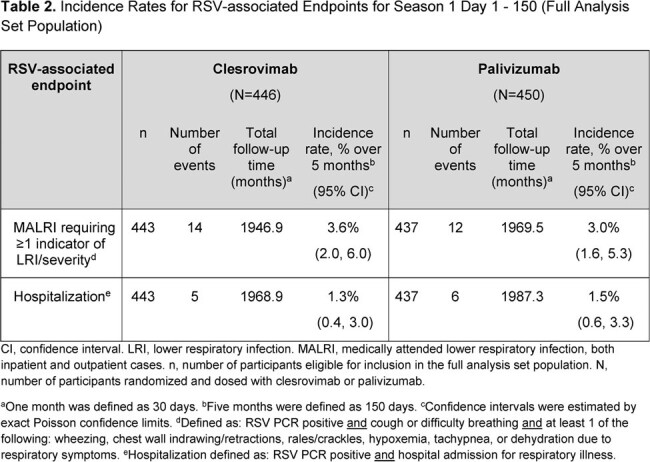

**Conclusion:**

Clesrovimab was well tolerated in infants at high risk for RSV disease. In season 1, a single dose of clesrovimab had a safety profile and RSV disease incidence rates that were generally comparable to monthly palivizumab.

**Figure:**
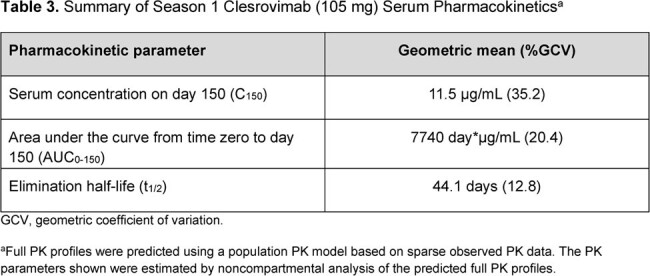

**Disclosures:**

**Heather J. Zar, PhD**, MSD, Pfizer, AstraZeneca, Moderna (DSMB): Advisor/Consultant|MSD, Pfizer, AstraZeneca, Moderna (DSMB): Grant/Research Support|MSD, Pfizer, AstraZeneca, Moderna (DSMB): Honoraria|MSD, Pfizer, AstraZeneca, Moderna (DSMB): MSD Principal Investigator for the study and on MSD Advisory Board **Louis J. Bont, MD**, Pfizer, AstraZeneca, Sanofi, Janssen, MeMed Diagnostics, Gates Foundation, GSK, Novavax, Julius Clinical, Ablynx, Bavaria Nordic, Moderna, and MSD: Advisor/Consultant|Pfizer, AstraZeneca, Sanofi, Janssen, MeMed Diagnostics, Gates Foundation, GSK, Novavax, Julius Clinical, Ablynx, Bavaria Nordic, Moderna, and MSD: Board Member|Pfizer, AstraZeneca, Sanofi, Janssen, MeMed Diagnostics, Gates Foundation, GSK, Novavax, Julius Clinical, Ablynx, Bavaria Nordic, Moderna, and MSD: Grant/Research Support|Pfizer, AstraZeneca, Sanofi, Janssen, MeMed Diagnostics, Gates Foundation, GSK, Novavax, Julius Clinical, Ablynx, Bavaria Nordic, Moderna, and MSD: Honoraria|Pfizer, AstraZeneca, Sanofi, Janssen, MeMed Diagnostics, Gates Foundation, GSK, Novavax, Julius Clinical, Ablynx, Bavaria Nordic, Moderna, and MSD: Regular interaction with pharmaceutical & other industrial partners.Founding chairman-ReSViNET Foundation.Received minor institutional funding to UMCU **Paolo Manzoni, MD, PhD**, Merck Sharp & Dohme LLC, a subsidiary of Merck & Co., Inc., Rahway, NJ, USA (MSD): Advisor/Consultant|Merck Sharp & Dohme LLC, a subsidiary of Merck & Co., Inc., Rahway, NJ, USA (MSD): SAC member for MSD **Flor M. Munoz, MD, MSc**, Pfizer, DMC-Moderna, Meissa, Sanofi, AstraZeneca, Novavax, Gilead, MSD: Advisor/Consultant|Pfizer, DMC-Moderna, Meissa, Sanofi, AstraZeneca, Novavax, Gilead, MSD: Grant/Research Support|Pfizer, DMC-Moderna, Meissa, Sanofi, AstraZeneca, Novavax, Gilead, MSD: SAC member for MSD **Octavio Ramilo, MD**, Pfizer, Sanofi, Gates Foundation, NIH, and Merck Sharp & Dohme LLC, a subsidiary of Merck & Co., Inc., Rahway, NJ, USA (MSD): Advisor/Consultant|Pfizer, Sanofi, Gates Foundation, NIH, and Merck Sharp & Dohme LLC, a subsidiary of Merck & Co., Inc., Rahway, NJ, USA (MSD): Grant/Research Support|Pfizer, Sanofi, Gates Foundation, NIH, and Merck Sharp & Dohme LLC, a subsidiary of Merck & Co., Inc., Rahway, NJ, USA (MSD): Honoraria|Pfizer, Sanofi, Gates Foundation, NIH, and Merck Sharp & Dohme LLC, a subsidiary of Merck & Co., Inc., Rahway, NJ, USA (MSD): SAC member for MSD **Po-Yen Chen, MD**, Merck Sharp & Dohme LLC, a subsidiary of Merck & Co., Inc., Rahway, NJ, USA (MSD): Princial Investigator for MSD **Jose M. Novoa Pizarro, MD**, Merck Sharp & Dohme LLC, a subsidiary of Merck & Co., Inc., Rahway, NJ, USA (MSD): Principal Investigator for the study for MSD **Gustavo A. Ordonez, MD**, Megalabs, Tecnoquimicas SA and MSD: Grant/Research Support|Megalabs, Tecnoquimicas SA and MSD: Honoraria|Megalabs, Tecnoquimicas SA and MSD: Principal Investigator for the study for MSD **Maria Tsolia, MD, PhD**, Merck Sharp & Dohme LLC, a subsidiary of Merck & Co., Inc., Rahway, NJ, USA (MSD): Princial Investigator for MSD **Bruce Tapiero, MD**, Merck Sharp & Dohme LLC, a subsidiary of Merck & Co., Inc., Rahway, NJ, USA (MSD): Princial Investigator for MSD **Mirta Acuña, MD**, Pfizer, Janssen, Sanofi, and MSD: Advisor/Consultant|Pfizer, Janssen, Sanofi, and MSD: Grant/Research Support|Pfizer, Janssen, Sanofi, and MSD: Principal Investigator for Janssen, Sanofi and MSD **Javier M. Castellanos, MD**, Merck Sharp & Dohme LLC, a subsidiary of Merck & Co., Inc., Rahway, NJ, USA (MSD): Princial Investigator for MSD **Michael Meyer, MD**, Merck Sharp & Dohme LLC, a subsidiary of Merck & Co., Inc., Rahway, NJ, USA (MSD): Princial Investigator for MSD **Ichiro Morioka, MD, PhD**, AstraZeneca K.K.: Honoraria|Merck Sharp & Dohme LLC, a subsidiary of Merck & Co., Inc., Rahway, NJ, USA (MSD): Honoraria|Merck Sharp & Dohme LLC, a subsidiary of Merck & Co., Inc., Rahway, NJ, USA (MSD): Princial Investigator for MSD|Mitsubishi Tanabe Pharma Corporation: Honoraria|Pfizer Japan Inc.: Honoraria|Sanofi K.K.: Honoraria|Shino-Test Corporation: Grant/Research Support **Ziqiang Chen, PhD**, Merck Sharp & Dohme LLC, a subsidiary of Merck & Co., Inc., Rahway, NJ, USA (MSD): Employee|Merck Sharp & Dohme LLC, a subsidiary of Merck & Co., Inc., Rahway, NJ, USA (MSD): Stocks/Bonds (Public Company) **Radha A. Railkar, PhD**, Merck Sharp & Dohme LLC, a subsidiary of Merck & Co., Inc., Rahway, NJ, US (MSD): Employee|Merck Sharp & Dohme LLC, a subsidiary of Merck & Co., Inc., Rahway, NJ, US (MSD): Stocks/Bonds (Public Company) **Xiaowei Zang, PhD**, Merck Sharp & Dohme LLC, a subsidiary of Merck & Co., Inc., Rahway, NJ, USA (MSD): Employee|Merck Sharp & Dohme LLC, a subsidiary of Merck & Co., Inc., Rahway, NJ, USA (MSD): Stocks/Bonds (Public Company) **Andrea L. Krick, PhD**, Merck Sharp & Dohme LLC, a subsidiary of Merck & Co., Inc., Rahway, NJ, USA (MSD): Employee|Merck Sharp & Dohme LLC, a subsidiary of Merck & Co., Inc., Rahway, NJ, USA (MSD): Stocks/Bonds (Public Company) **Andrew W. Lee, MD**, Merck Sharp & Dohme LLC, a subsidiary of Merck & Co., Inc., Rahway, NJ, USA (MSD): Employee at the time of study|Merck Sharp & Dohme LLC, a subsidiary of Merck & Co., Inc., Rahway, NJ, USA (MSD): Stocks/Bonds (Public Company) **Luis A. Castagnini, MD, MPH**, Merck Sharp & Dohme LLC, a subsidiary of Merck & Co., Inc., Rahway, NJ, USA (MSD): Employee|Merck Sharp & Dohme LLC, a subsidiary of Merck & Co., Inc., Rahway, NJ, USA (MSD): Stocks/Bonds (Public Company) **Anushua Sinha, MD, MPH**, Merck Sharp & Dohme LLC, a subsidiary of Merck & Co., Inc., Rahway, NJ, USA (MSD): Employee|Merck Sharp & Dohme LLC, a subsidiary of Merck & Co., Inc., Rahway, NJ, USA (MSD): Stocks/Bonds (Public Company)

